# Supplementary material for: Adaptive laboratory evolution recruits the promiscuity of succinate semialdehyde dehydrogenase to repair different metabolic deficiencies
Source: Nat Commun. 2024 Oct 15;15:8898. doi: 10.1038/s41467-024-53156-x (PMC11480449; doi:10.1038/s41467-024-53156-x)
Supplement: Supplementary file 1 — Supplementary Information [file 41467_2024_53156_MOESM1_ESM.pdf]

Adaptive laboratory evolution recruits the promiscuity of succinate semialdehyde dehydrogenase to repair different metabolic deficiencies

He *et al.*

Supplementary Note 1. SIJ488 genome *de novo* assembly

It is known that *E. coli* K-12 MG1655 strains from different collections have slight genomic differences. We noticed that the SIJ488 strain[1] has different genomic sites than the reference MG1655 genome (GenBank: U00096 or NC_000913)[2] other than the integrated λRed system. Therefore, we sequenced the genome of SIJ488 *via* Illumina and Nanopore techniques and assembled the genome.

For Illumina sequencing, NucleoSpin Microbial DNA kit (MACHERY-NAGEL, Düren, Germany) was used for genomic DNA extraction following manufacturer's instructions. Library construction used Nextera XT kit (Illumina) and genome sequencing was on a paired-end Illumina sequencing platform MiSeq. For Nanopore sequencing, genomic DNA of stationary phase cells was obtained using the NucleoBond HMW DNA kit (Macherey-Nagel, Düren, Germany) according to the manufacturer guidelines, and using lysozyme for cell lysis (final concentration: 1 mg/mL) for 1 h at 37 °C in 2 mL of 10 mM Tris-HCl, pH 8.0. Sequencing was performed on a MinION Mk1B device using a MinION Flow Cell (FLO-MIN111, cell chemistry R10.1). Nanopore data was base-called with ONT Guppy base-calling software (v 6.0.1+652ffd179). Raw reads are deposited at the NCBI Sequence Read Archive (SRA) and can be accessed under BioProject PRJNA1002757.

Assembly of the genome followed the pipeline of MicroPIPE[3] with changes. The Nanopore raw data was trimmed by Porechop (v0.2.4) and filtrate by Filtlong (v0.2.1), then assembled by Flye (v2.8.1)[4]. The resulting sequence was then polished by medaka (v0.7.1) and NextPolish (v1.4.1) using Nanopore data and Illumina data, respectively. The final assembled genome (4,648,169 bp) was manually set +1 locus to the same as MG1655 and deposited at NCBI GenBank and can be accessed under CP132594. The differences between SIJ488 genome and MG1655 genome were compared using RATT[5] (https://github.com/ThomasDOtto/ratt) and manual curation, can be found on Edmond at https://doi.org/10.17617/3.6JLOFX.

Supplementary Note 2. P3Pe mutations

The evolved P3Pe together with its parental glycerate auxotroph strain P3P (Supplementary Table 1) were subjected to short-reads whole genome sequencing. Mutations identified are listed in Supplementary Table 6, revealing changes in P3Pe strain compared to its parental P3P strain during ALE. The changes are a key mutation E97A in the *sad* operon transcription factor YneJ, and the other three mutations that seem to be irrelevant to the phenotype. Knock out of the engineered *pccA* and *pccB* genes from the plasmid, which is for glycolyl-CoA carboxylase of the TaCo pathway[6], would only result in an incomplete pathway. A385V substitution in phosphoglycerate dehydrogenase (SerA), which catalyzes the first step in serine biosynthesis, locates in the allosteric regulatory ACT domain (Supplementary Figure 13a). As it has been reported that mutations in the adjacent residues D386A and E387A or even removing the whole ACT domain did not significant alter enzyme catalytic[7,8], A385V mutation is unlikely to relieve the be responsible for selection bypassing. Finally, *insB1-insA* is a transposon element and its knock out is often found in ALE.

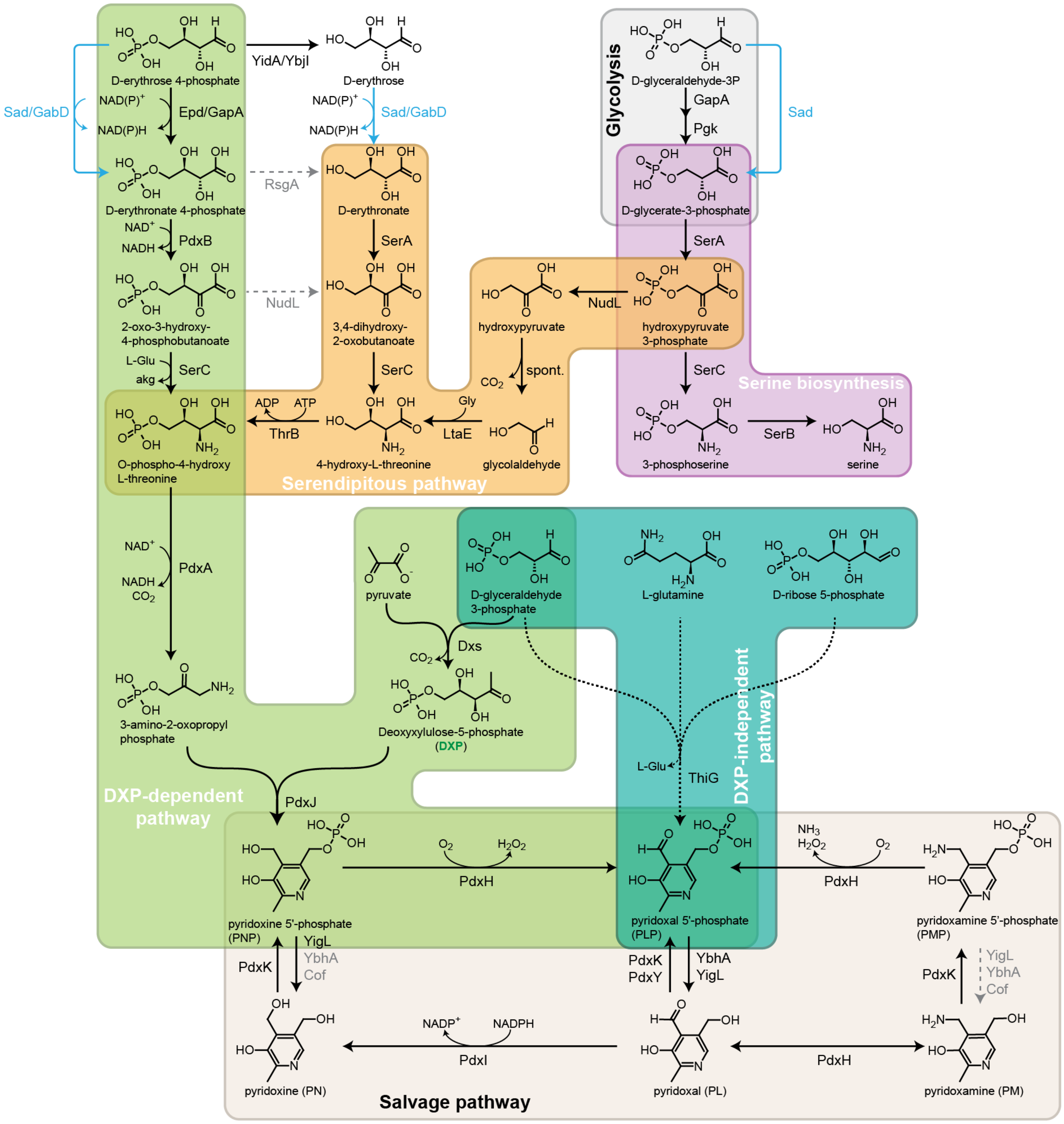

Supplementary Figure 1. Metabolic network of pyridoxal 5'-phosphate biosynthesis in *E. coli*.

Biosynthesis from erythrose 4-phosphate is the major pathway (the DXP-dependent pathway), and the 4-hydroxy-L-threonine route is a serendipitous pathway. While the PLP synthase route (DXP-independent pathway) was thought to be absent in *E. coli*, ThiG, thiazole synthase (dotted reaction), was proposed to promiscuously have such activity[9]. Reactions in blue are confirmed in this study. Reactions in dashed grey are proposed, in which RsgA was proposed by Richts and Commichau[10].

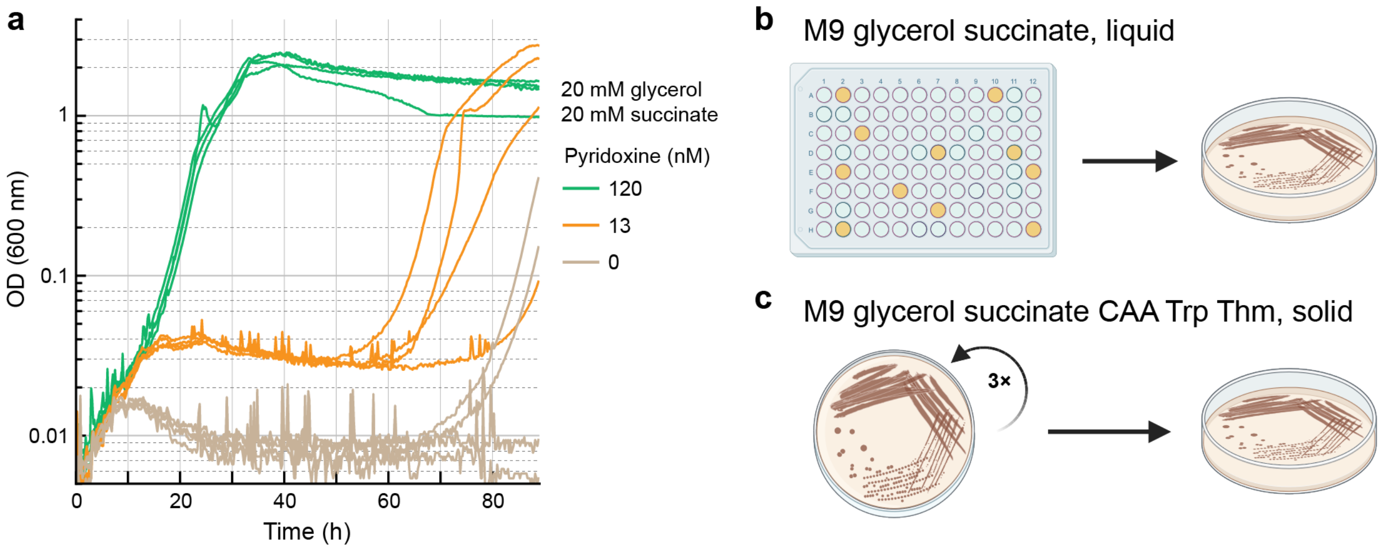

Supplementary Figure 2. Adaptive laboratory evolution of Δ*epd* Δ*gapA* strains.

(**a**) Growth curves showcase evolution of strain EG2 (Supplementary Table 1) in a short time. Under conditions where pyridoxine was absent or at low concentrations, some replicates of the culture emerged to grow after 50 h. (**b**) Such culture was streaked onto plates to isolate single colonies of mutations, resulting in strains EG1.1, EG1.2x, EG2.1t, and EG2.2s. (**c**) Separately, ALE was conducted on solid medium with casamino acids (CAA), tryptophan (Trp) and thiamine (Thm), obtaining strains EG1.3, EG1.4, EG1.5, EG2.3a, EG2.4a, and EG2.5a. See Methods for details. Panels **b** and **c** were created in BioRender. He, H. (2023) BioRender.com/p39e931. Source data are provided as a Source Data file.

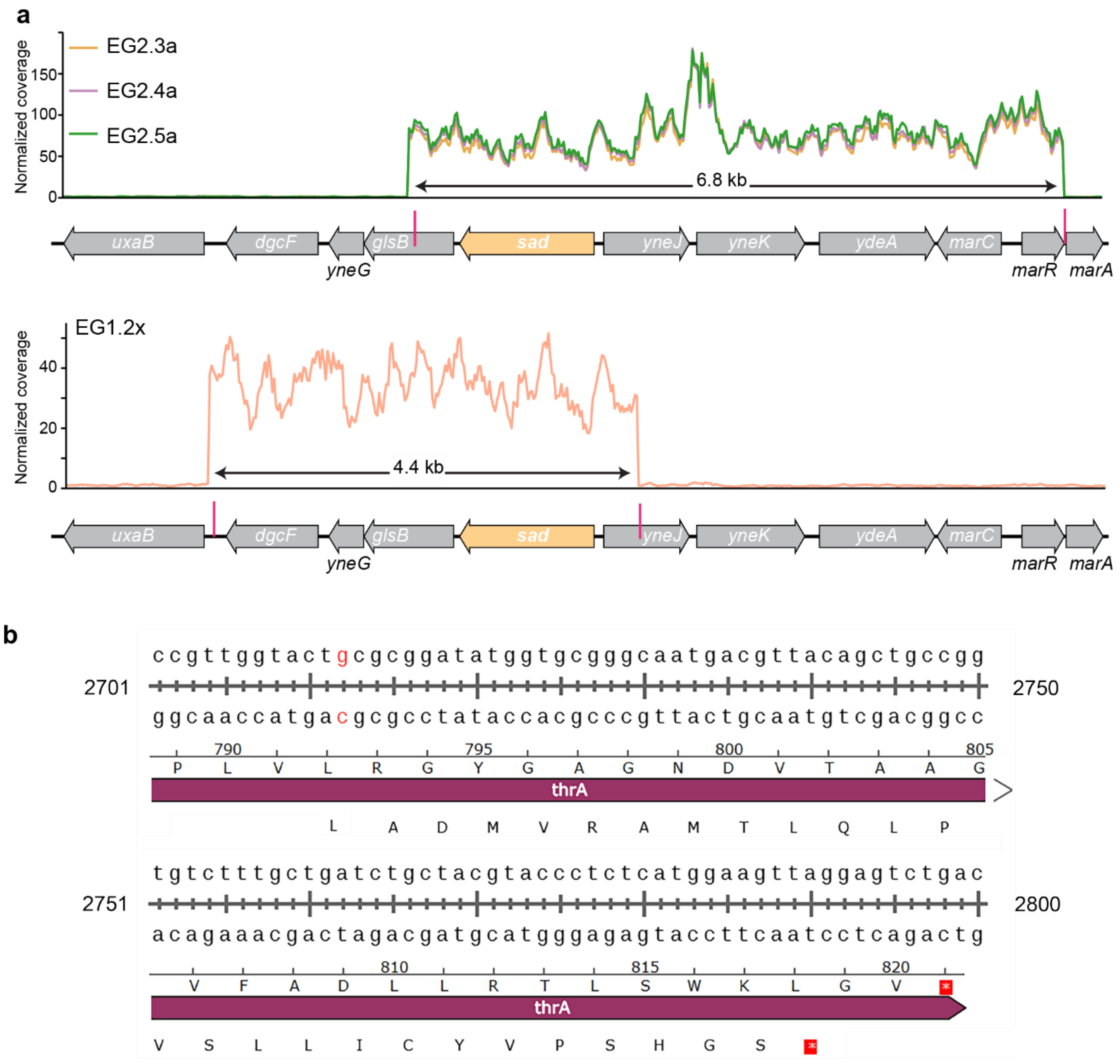

Supplementary Figure 3. Amplification of *sad* and frameshift of *thrA*.

(**a**) Genomic context of *sad* in *E. coli* coverage change of amplified region. *breseq*[11] predicted "New Junction" in EG2.3a, EG2.4a, EG2.5a, and EG1.2x strains (Supplementary Table 1 and 2) at the points marked with the pink lines. From the type of "New Junction" and increased coverage, we manually annotated the regions in between the pink lines "gene amplification" (tandem repeat). The line plots show the normalized coverage from *breseq* mapping. Approximately 80× and 30× increases of coverage of the amplified regions were estimated. Coverage statistics are detailed in Supplementary Table 3. (**b**) Frameshift of *thrA* in EG1.2t. A base deletion, G2712 (highlighted in red), caused frameshift and the reading frame changed to +2 resulting amino acids shown below. Genomic location coordinates are shown on the side. Source data are provided as a Source Data file.

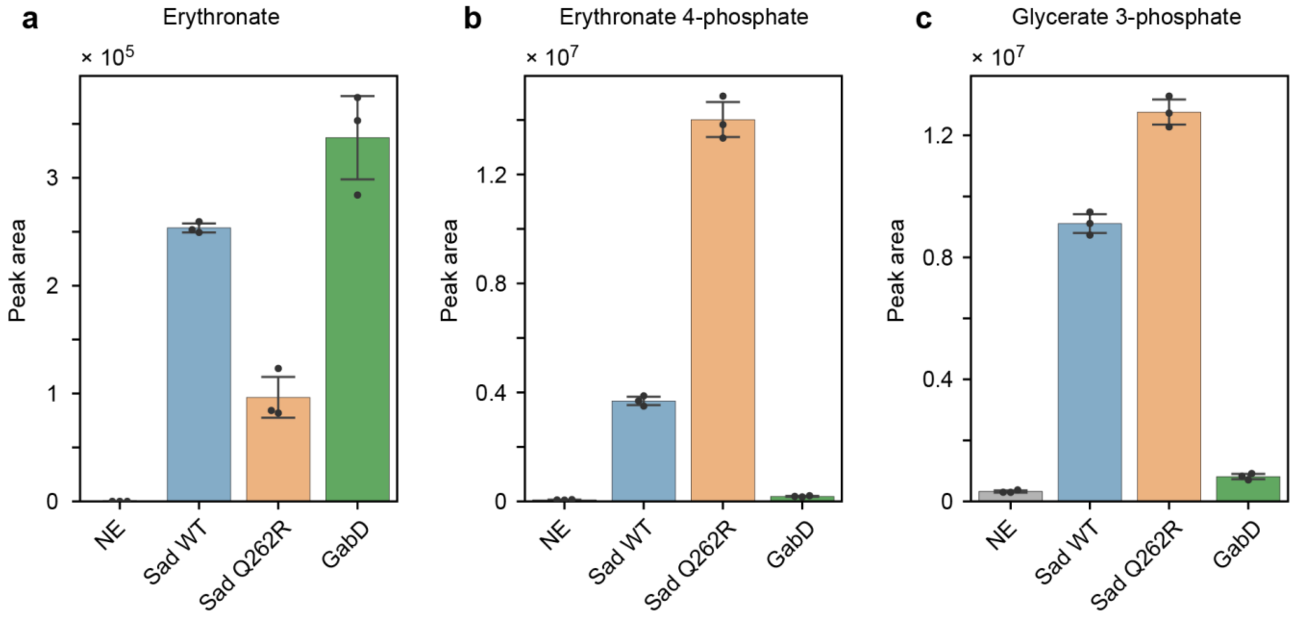

Supplementary Figure 4. LC-MS confirmation of the oxidation reaction of SSADHs on non-primary substrates.

In vitro reaction products of Sad WT, Sad Q262R and GabD on erythrose, erythrose 4-phosphate and glyceraldehyde 3-phosphate were subjected to LC-MS analysis (see Methods). Panels present peak area of their corresponding erythronate (**a**), erythronate 4-phosphate (**b**) and glycerate 3-phosphate (**c**). NE represents no enzyme control. Measurements show that the substrate chemicals contain impurities including their corresponding oxidized compound. Error bars represent SD, N = 3. Source data are provided as a Source Data file.

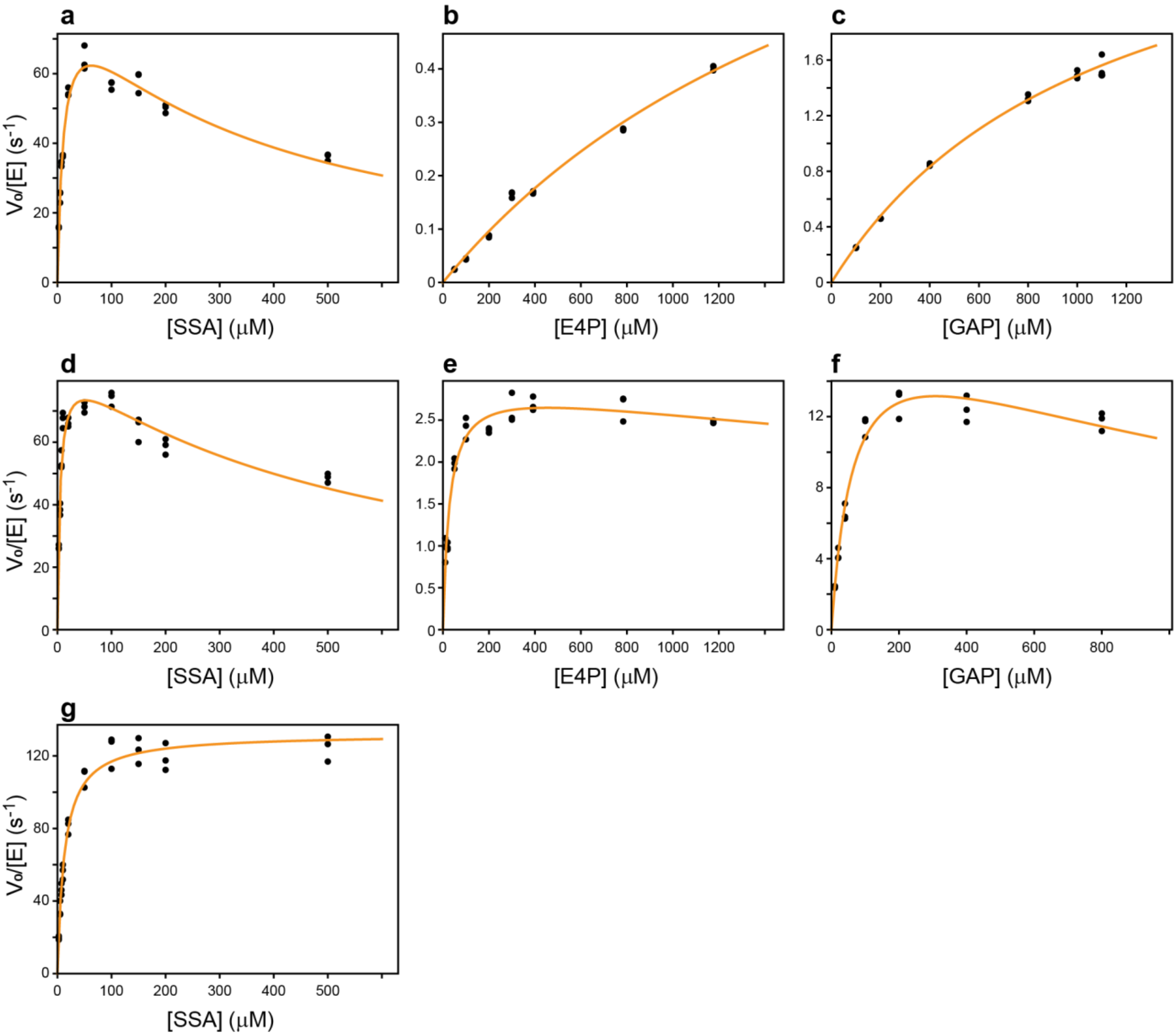

Supplementary Figure 5. Kinetic analysis of SSADHs.

(**a**), (**b**) and (**c**) are Sad WT; (**d**), (**e**) and (**f**) are Sad Q262R; and (**g**) is GabD. The reactions were carried out in 75 mM HEPES pH 8.0, 37 °C in the present of 1 mM NAD^+, or $NADP^+$ in the case of GabD, as described in Methods. The plots were fitted to the equation of the Michaelis-Menten mechanism, $V_0/[\mathrm{E}] = V_{max}[S]/(K_m + [S])$, or substrate inhibition, $V_0/[\mathrm{E}] = V_{max}[S]/(K_m + [S](1 + [S]/K_i))$, by nonlinear regression using python scripts, and the K_m and k_cat of SSADHs for each substrate were calculated. N = 3. E4P – erythrose 4-phosphate; GAP – glyceraldehyde 3-phosphate; SSA – succinate semialdehyde. Source data are provided as a Source Data file.

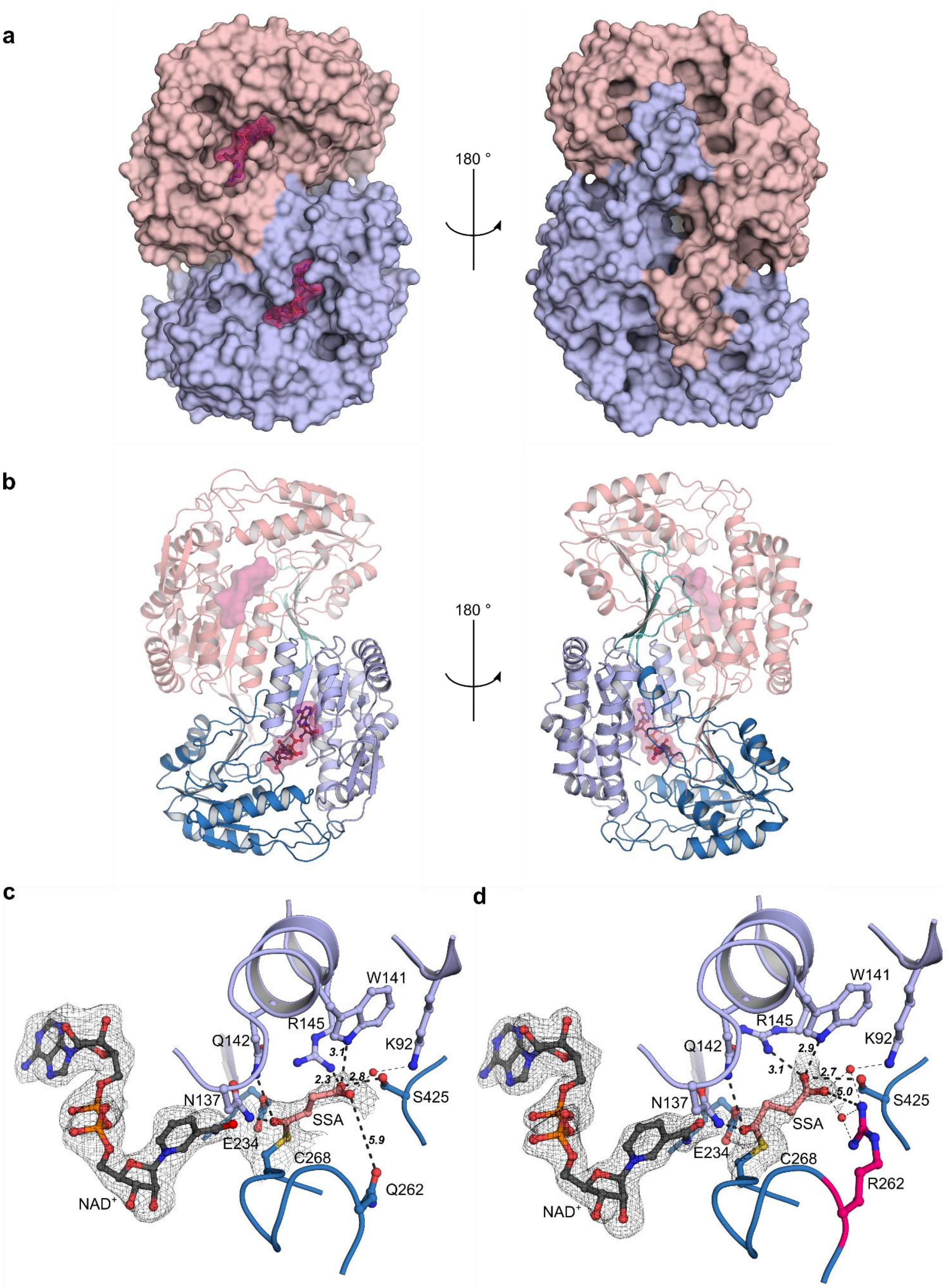

Supplementary Figure 6. Crystal structure of Sad in complex with NAD^+ and succinate semialdehyde (SSA).

(**a**) Surface of the Sad dimer. Protein subunits are shown in salmon and light blue. Bound substrates, NAD^+ and SSA are shown in pink. (**b**) Cartoon representation of the Sad structure. Three different domains are shown for the lower subunit: the NAD^+-binding domain in light blue, the catalytic domain in dark blue, and the oligomerization domain in cyan. (**c**) The active sites of the wild-type enzyme with bound NAD^+ (dark grey) and SSA (salmon). A simulated annealing omit map (*Fo-Fc)* is depicted for the substrates and the catalytic Cys268 at 1.8 σ showing covalent bonding between SSA and C268. The hydrogen bonding network for the coordination of SSA is shown as black dashed lines. Important distances are given in Å. Potential interactions with coordinating water molecules are represented by thinner black dashed lines. (**d**) The active site of the Q262R variant of Sad with bound NAD^+ and SSA. A simulated annealing omit map (*Fo-Fc)* is depicted for the substrates and the catalytic Cys268 at 2.0 σ. Coloring is as in (**c**), with the Q262R substitution highlighted in pink.

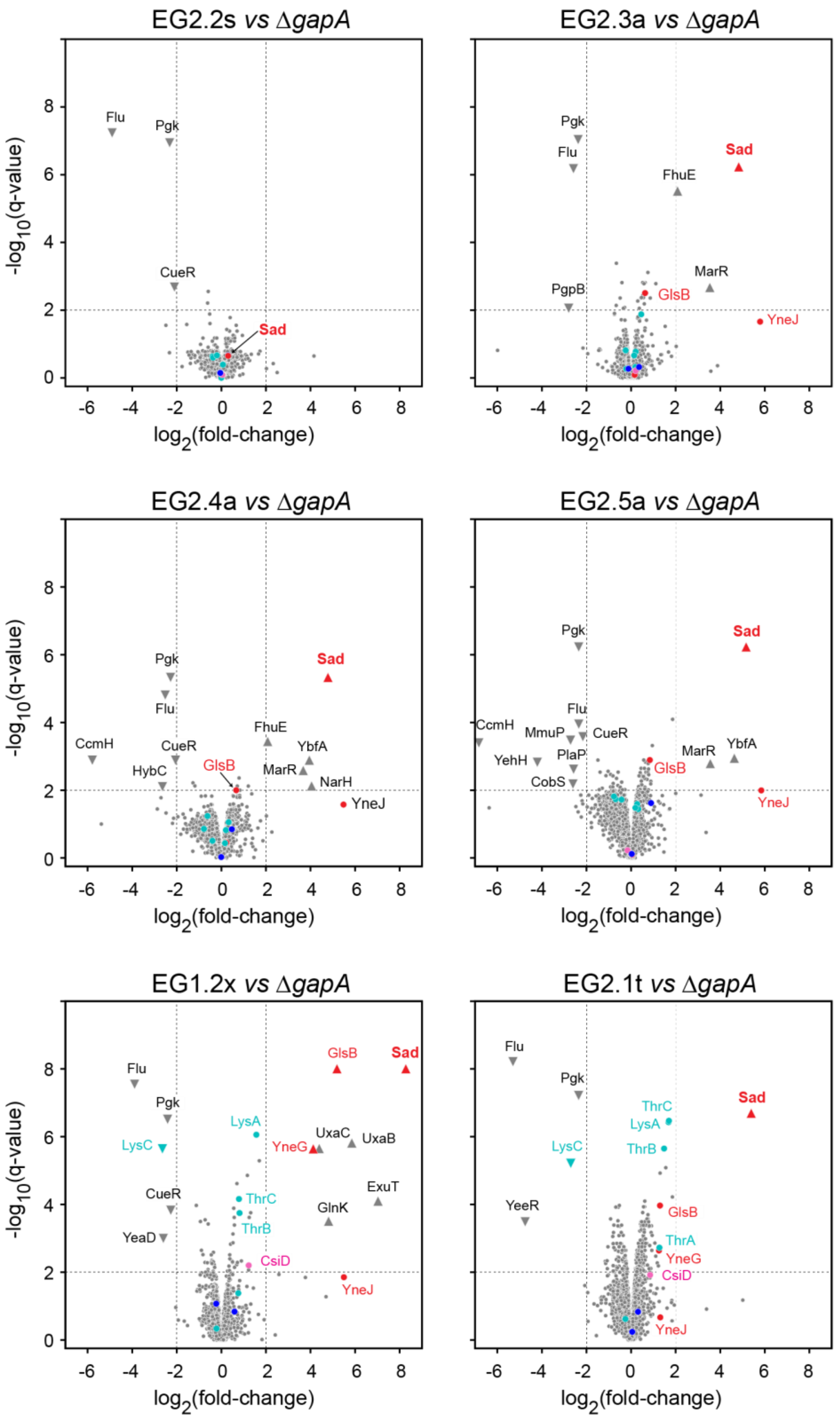

Supplementary Figure 7. Volcano plots of relative proteome in Δ*epd* Δ*gapA* evolved strains.

The fold-change ratio represents the mean intensity relative to the Δ*gapA* strain mean intensity. The Y-axis indicates the $-\log_{10}$(q-value), where q-value is the adjusted p-value according to the Benjamini-Hochberg method. All strains were cultured on M9 5 mM glycerol, 20 mM succinate without pyridoxine and harvested at the exponential phase. N = 4. All proteins that increased or decreased significantly (q-value < 0.01) by at least 4-fold are marked with upward and downward triangles, respectively. The *sad* gene operon products (YneJ, Sad, GlsB and YneG) are highlighted in red; the selected aspartate-deriving amino acids biosynthesis proteins (ThrA, ThrB, ThrC, LysC, LysA, and MetL) are in cyan; the two E4P phosphatases (YidA and YbjI) are in blue; and CsiD is in pink.

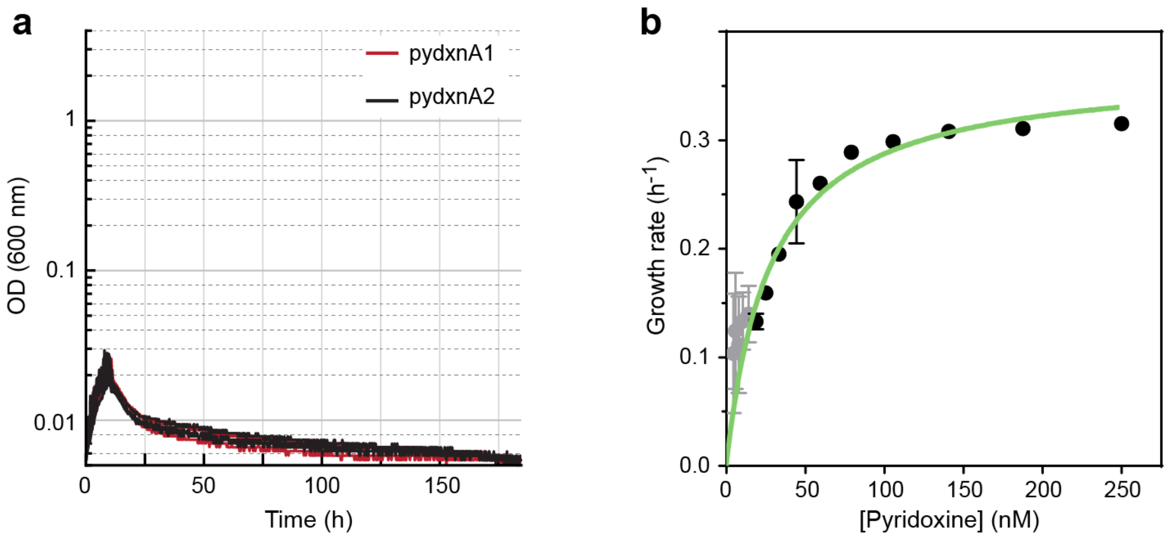

Supplementary Figure 8. The strain Δ*epd* Δ*thrB* Δ*gabD* Δ*sad* Δ*gapA* is tight and sensitive to pyridoxine.

(**a**) The new knock-out strains were not able to grow on minimal medium without pyridoxine (M9 5 mM glycerol, 20 mM succinate, 0.5 mM threonine) within a week. pydxnA1 and pydxnA2 are biological replicate strains, each has 4 technical replicate cultures. Their growth under gradient concentrations of pyridoxine is shown in Fig. 4c. (**b**) Growth rate in function of pyridoxine concentration in medium. Growth rates, μ, were fitted to the Michaelis-Menten equation, $\mu = \mu_{max}[S]/(K_m + [S])$, by nonlinear regression, and K_m and μ_{max} were calculated to be 28 nM and 0.37 h^{-1}, respectively. Noisy growth rates corresponding to [pyridoxine] below 15 nM were excluded in data fitting and shown in grey. N = 6, error bars represent SD. Source data are provided as a Source Data file.

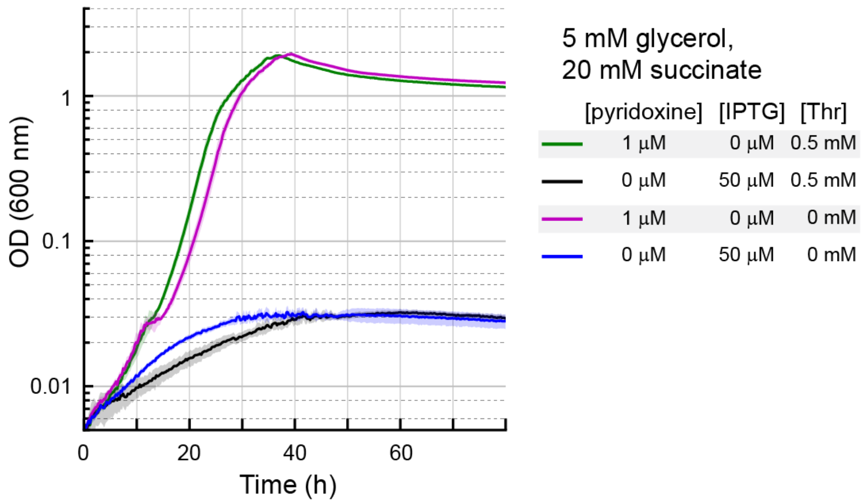

Supplementary Figure 9. ThrB overexpression restores growth of pydxnA strain only in the presence of pyridoxine.

The pydxnA strain (Supplementary Table 1) is auxotrophic to both pyridoxine and threonine. Overexpressing ThrB from a plasmid (pTE3858, Supplementary Table 5) relieved auxotrophy of threonine but not pyridoxine. Lines represent mean values and patches show SD, N = 4. Source data are provided as a Source Data file.

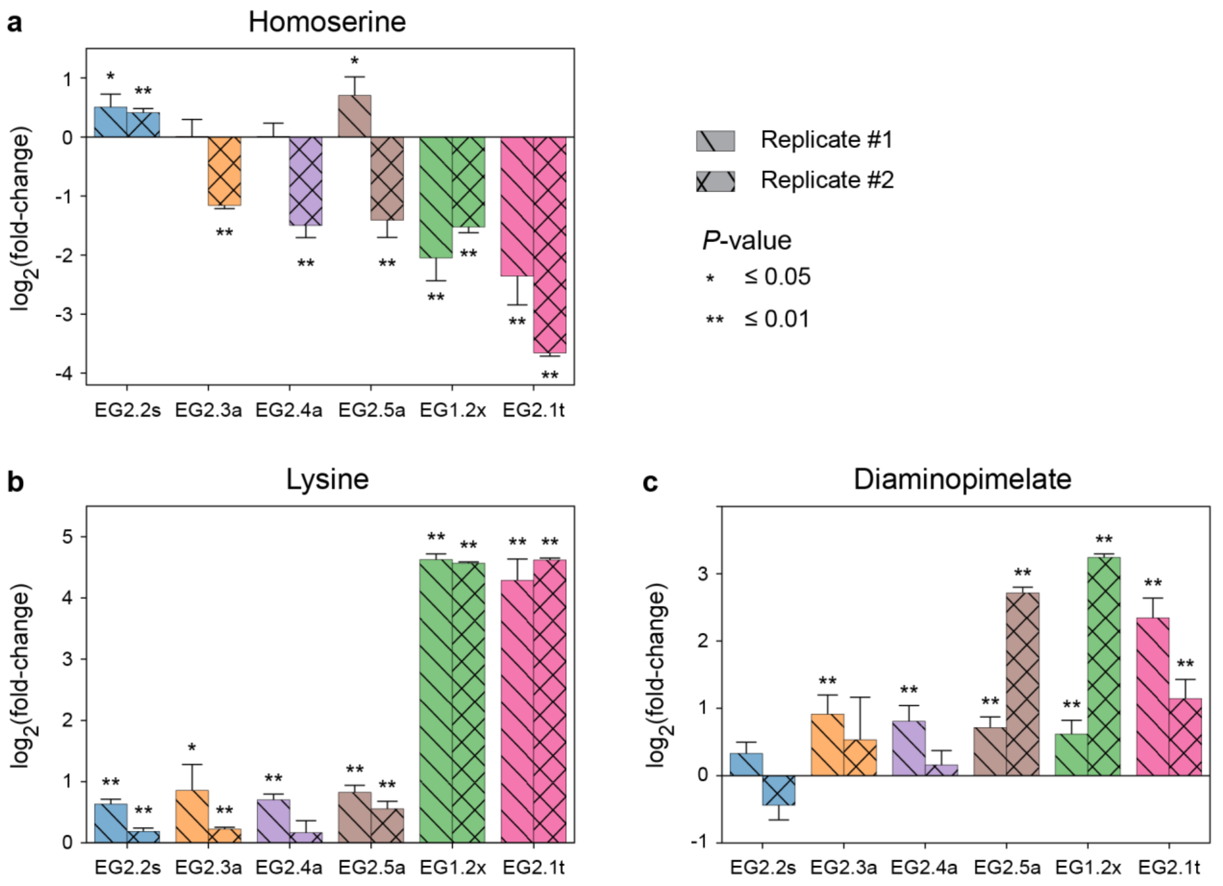

Supplementary Figure 10. Endometabolomic profiles of Δ*epd* Δ*gapA* evolved strains.

The fold-change ratios are relative to Δ*gapA* strain. Error bars represent SD, N = 4. All strains were cultured on M9 5 mM glycerol, 20 mM succinate without pyridoxine and harvested at late exponential phase. Significant differences of metabolite intracellular concentrations compared to Δ*gapA* strain are indicated by * (*P*-value ≤ 0.05) or ** (*P*-value ≤ 0.01), Two-tailed T-test. Two independent replicated experiments were conducted. While variations were observed between the two replicates, homoserine concentrations decreased constantly within the *thrA* mutant strain EG1.2x and EG2.1t (**a**). Lysine concentrations increased remarkably, ~ 20-fold, in these strains in both replicates (**b**). On the other hand, the changes in diaminopimelate varied greatly between replicates (**c**). Together, it implies that lysine, rather than diaminopelate, is the effector molecule. Source data are provided as a Source Data file.

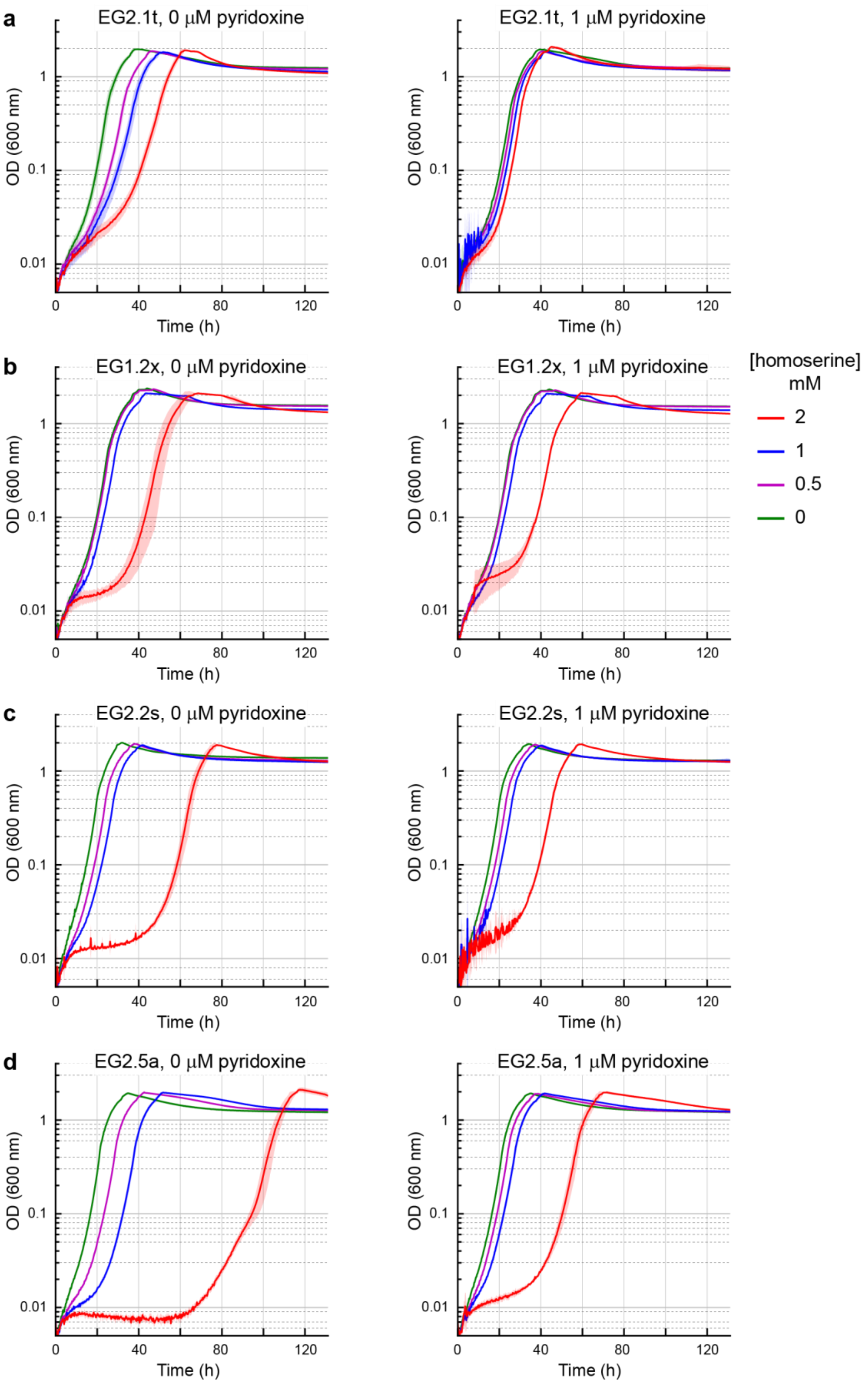

Supplementary Figure 11. Homoserine is inhibitory to the mutation strains even in the presence of pyridoxine.

Strains EG2.1t (**a**), EG1.2x (**b**), EG2.2s (**c**) and EG2.5a (**d**) grew on 20 mM succinate, 5 mM glycerol, with (right) or without (left) 1 μM pyridoxine and gradient concentrations of homoserine. Lines represent mean values and patches show SD, N = 3. Source data are provided as a Source Data file.

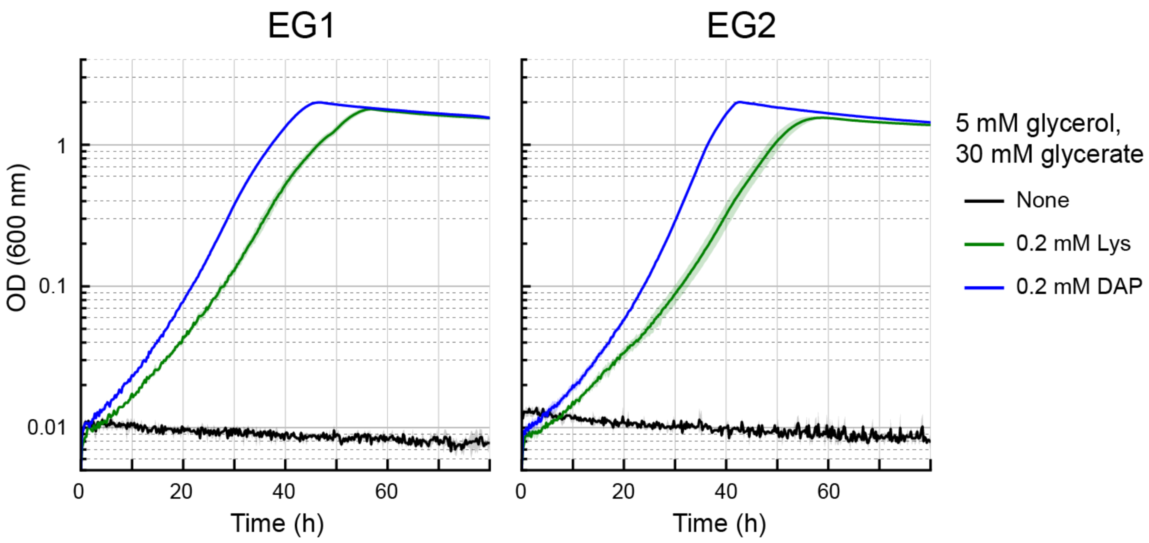

Supplementary Figure 12. Supplementing lysine (Lys) or diaminopimelate (DAP) enabled growth of Δ*epd* Δ*gapA*.

While only on glycerate, not on succinate, Lys or DAP at 0.2 mM in the medium restored growth of the Δ*epd* Δ*gapA* strains, EG1 and EG2. Source data are provided as a Source Data file.

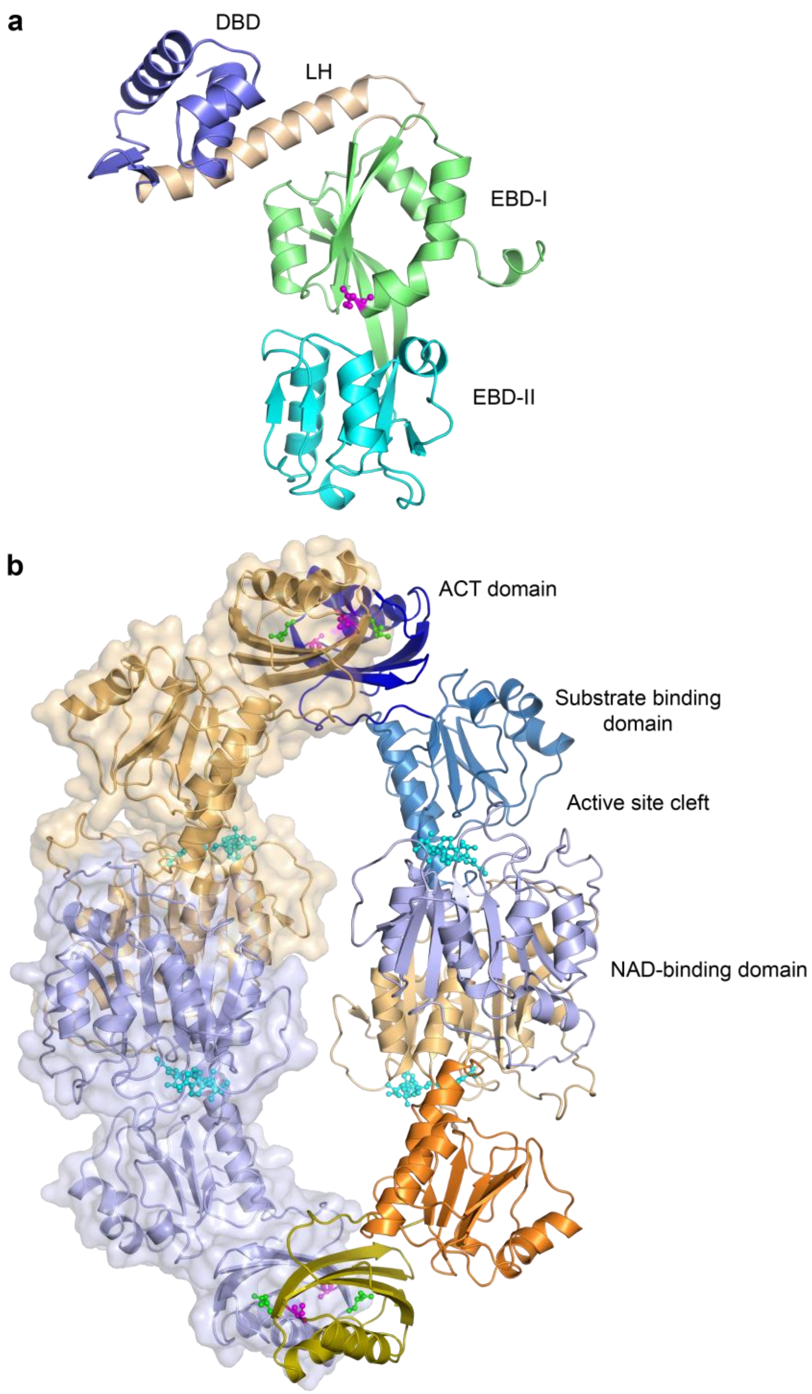

Supplementary Figure 13. Mutations in SerA and YneJ locate within the regulatory domains.

(**a**) Cartoon representation of the YneJ structure. The structure model is from the AlphaFold DB (https://alphafold.ebi.ac.uk)[12,13], entry P77309, with the model confidence average pLDDT of 90.8. Protein domains[14,15] are shown in different colors: the DNA-binding domain (DBD) in blue, the linker helix (LH) in orange, the effector-binding domain (EBD) EBD-I in green, and EBD-II in cyan. The mutation residue of E97 is shown as ball-and-stick in magenta. (**b**) Cartoon representation of the SerA structure (1PSD[16]). Subunits of the homotetramer are colored in blue and orange, half (dimer) showing surface and the other half showing enzyme domains. The three domains are shaded differently: the NAD-binding domain is light, the substrate binding domain is medium, and the ACT domain is dark. The NAD cofactors (cyan), the allosteric serine (green), and the mutation residue of A385 (magenta) are shown as ball-and-stick models.

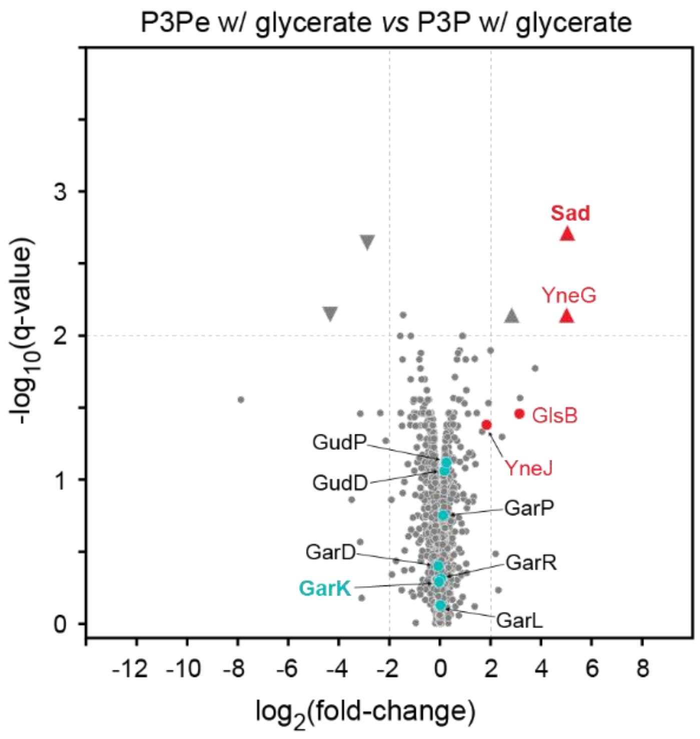

Supplementary Figure 14. Volcano plot of relative proteome in P3Pe strain.

The fold-change ratio is mean intensity relative to P3P strain mean intensity. The Y-axis indicates the $-\log_{10}$(q-value), where q-value is the adjusted *P*-value according to the Benjamini-Hochberg method. Both strains were cultured on M9 10 mM glycerol, 10 mM succinate 10 mM acetate, 4 mM glycine and 5 mM glycerate. N = 2. All proteins that increased or decreased significantly (q-value < 0.01) by at least 4-folds are marked with upward and downward triangles, respectively. The *sad* gene operon products (YneJ, Sad, GlsB and YneG) are highlighted in red; glycerate inducing proteins (GarPLRK, GarD, and GudPD)[17] are in cyan.

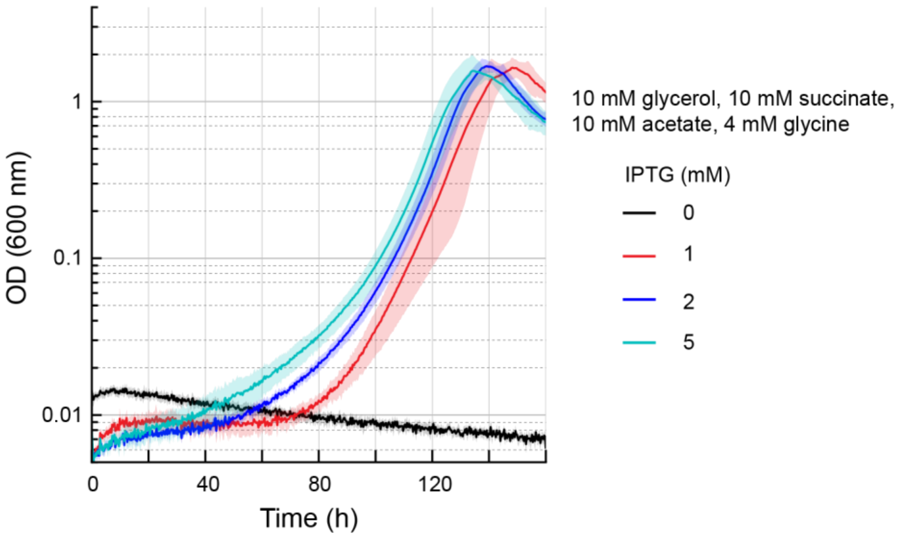

Supplementary Figure 15. Complement growth of strain P3Pe Δ*sad* required high IPTG induction for plasmid-based *sad* overexpression.

Strain P3Pe Δ*sad* (Supplementary Table 1) with plasmid pTE3827 (*sad* under $P_{LlacO\text{-}1}$ promoter, Supplementary Table 5) required high IPTG induction for *sad* overexpression to carry sufficient flux. No induction or low induction resulted no growth or long lag phase. Source data are provided as a Source Data file.

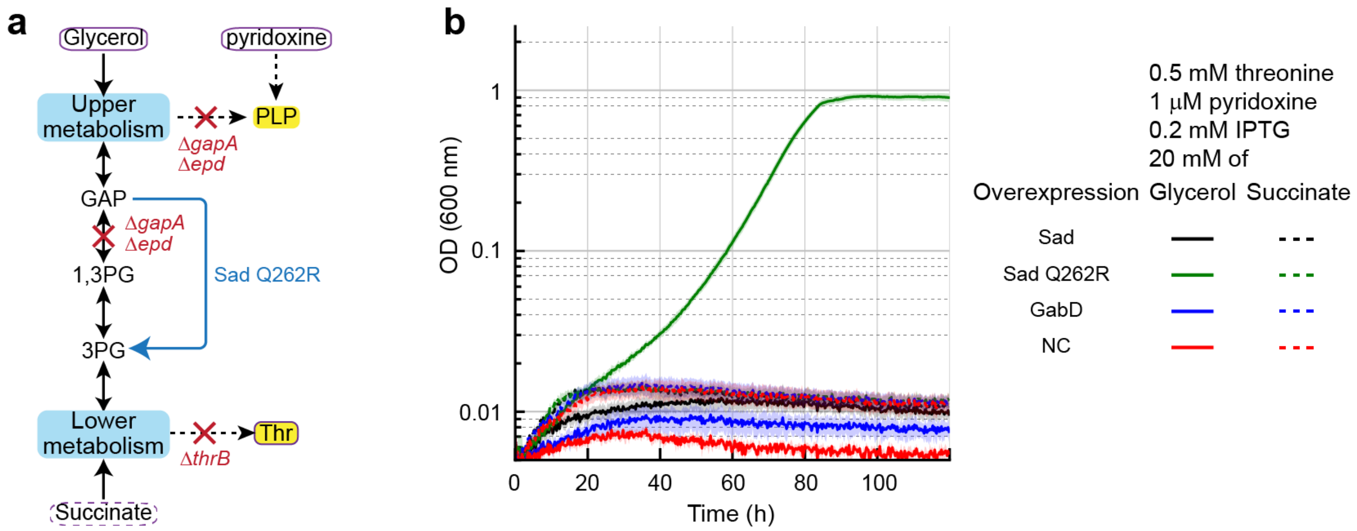

Supplementary Figure 16. Overexpression of Sad Q262R complements GAPDH for growth of pydxnA strain.

(**a**) Metabolic scheme of the pydxnA strain. Gene knock-outs are shown in red crosses. They result in pyridoxal 5'-phosphate (PLP) and threonine (Thr) auxotrophy. 1,3PG – glycerate 1,3-bisphosphate; 3PG – glycerate 3-phosphate; GAP – glyceraldehyde 3-phosphate. (**b**) When overexpressed from a plasmid, Sad Q262R can restore growth of pydxnA1 strain on glycerol (threonine and pyridoxine as well) but not on succinate, indicating Sad Q262R can efficiently convert GAP to 3PG irreversibly. This is in line with its thermodynamics, the non-phosphorylating GAPDH reaction has a $\Delta_r G'^m$ = 48 kJ/mol (calculated by eQuilibrator[18]). Sad WT and GabD were not able to rescue growth. N = 4, SD is shown in patch. Source data are provided as a Source Data file.

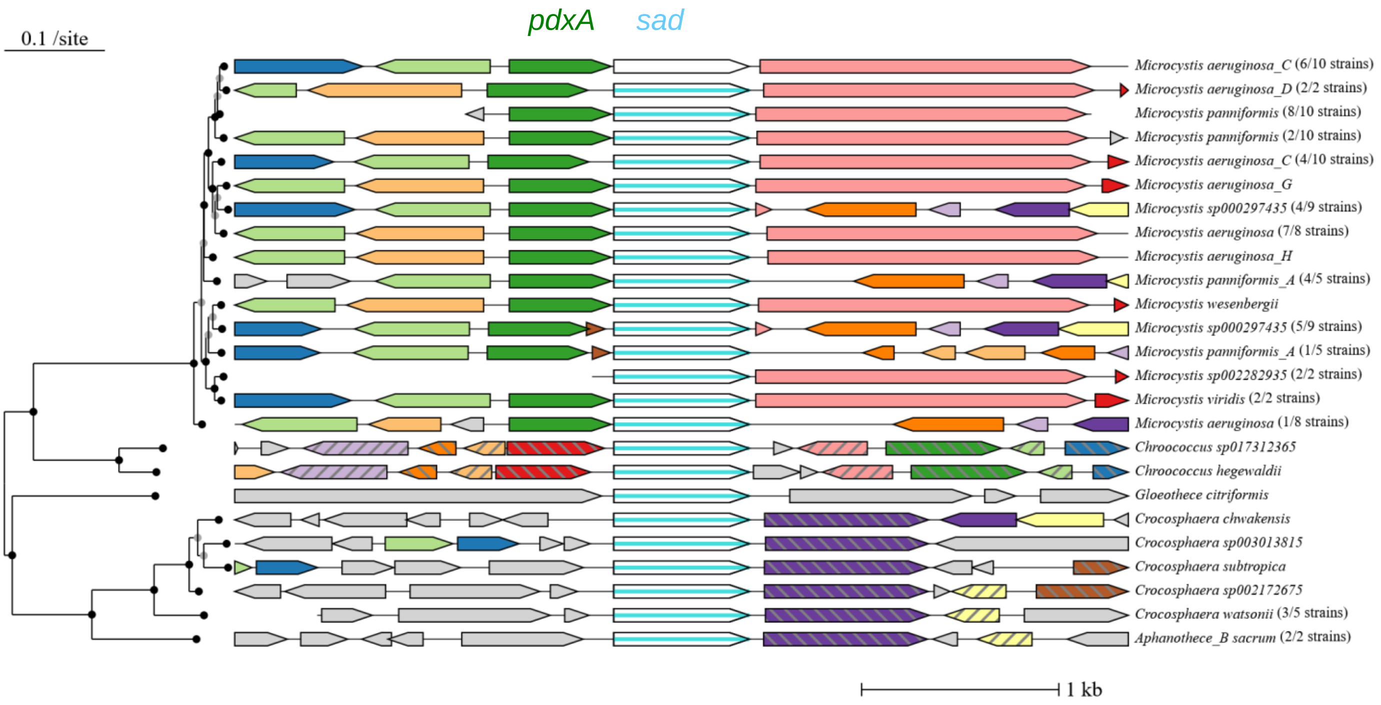

Supplementary Figure 17. Physical clustering PLP synthesis gene *pdxA* and *sad* genes in *Microcystis* genomes.

The Uniprot[19] for the predicted succinate-semialdehyde dehydrogenase [$NADP^+$] encoded by the model cyanobacteria *Synechocystis* sp. strain PCC 6803 (Q55585) was used as input in the fast.genomics[20] webserver tool (https://fast.genomics.lbl.gov/cgi/search.cgi). Using the "gene neighborhoods" tool with an expansion d to 200 genomes with default parameters showed that *sad* genes were located just downstream *pdxA* genes in several *Microcystis* genomes. Using one of the those genes (myaer_RS05540) as input in fast.genomics, the gene neighborhood tool was used changing the number of genome to 25, the region covered to 9 kb and adding the tree view feature led to the figure above with *pdxA* genes in green and *sad* genes in light blue. Note that although two homologs of GAPDH (EC 1.2.1.59 and EC 1.2.1.12) were found in the genomes of *Microcystis aeruginosa*, Epd (and GapN) was not identified.

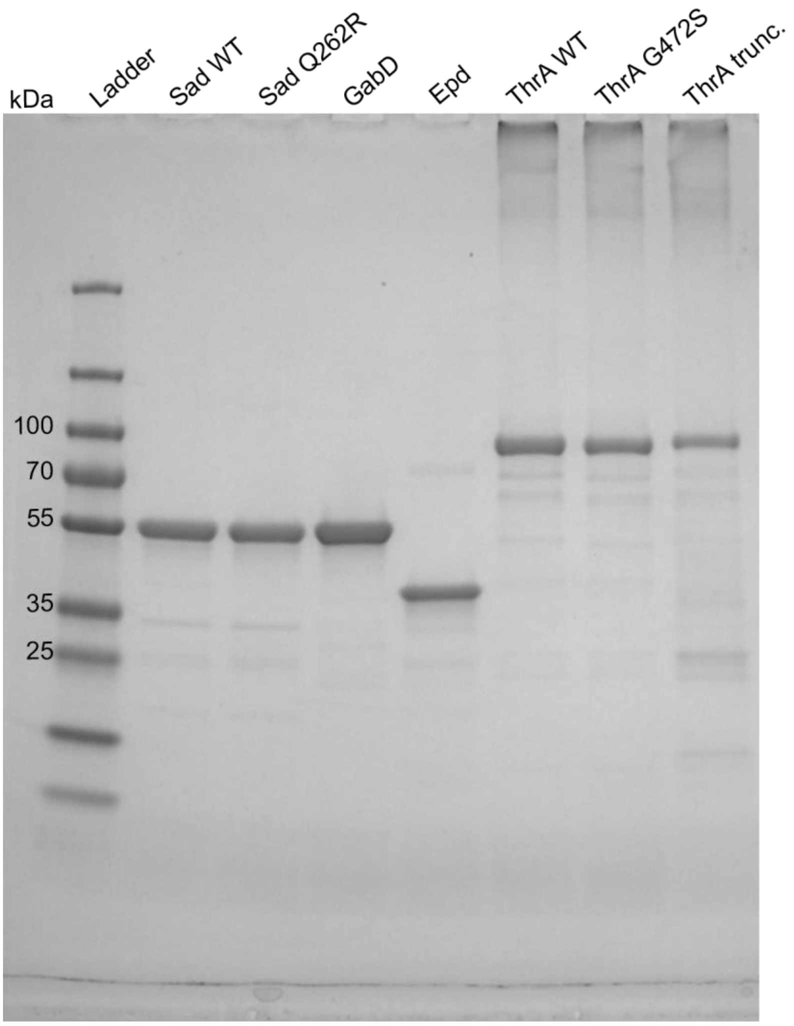

Supplementary Figure 18. SDS-PAGE gel of purified recombinant proteins.

2.5 μg of proteins were denatured in 2× loading buffer (from 5× Non-reducing lane marker sample buffer with 100 mM DTT, Thermo), then loaded to a SDS-PAGE gel (4-20%, Mini-PROTEAN TGX precast gel, Bio-Rad, USA). The prestained protein ladder, 5 μL, is from Thermo (PageRuler plus). The gel was stained with GelCode Blue Safe Protein Stain (Thermo). Source data are provided as a Source Data.

Supplementary Table 1. List of strains used in the study.

Strain	Genotype	Source
MG1655	K-12 $F^-\lambda^-$ *ilvG*$^-$*rfb*-50 *rph*-1	Lab collection
SIJ488	MG1655 Tn7::para-exo-beta-gam; prha-FLP; xylSpm-IsceI	[1]
DH5α	F^- *endA1 glnV44 thi-1 recA1 relA1 gyrA96 deoR nupG purB20* φ80dlacZΔM15 Δ(lacZYA-argF)U169, hsdR17($r_K^- m_K^+$), λ^-	Lab collection
Pir1	F- Δ*lac169 rpoS(Am) robA1 creC510 hsdR514 endA recA1 uidA(ΔMluI)::pir-116*	Invitrogen
BL21(DE3)	F– *omp*T *hsdS*$_B$ (r_B–, m_B–) *gal dcm* (DE3)	Lab collection
Δ*epd*	SIJ488 Δ*epd::Km*	This study
Δ*gapA*	SIJ488 Δ*gapA::CAP*	This study
EG1	SIJ488 Δ*epd::Km* Δ*gapA::CAP*, isolate E7	This study
EG2	SIJ488 Δ*epd::Km* Δ*gapA::CAP*, isolate E8	This study
EG1.1	EG1 mutant grew on M9 without pyridoxine, isolate E7A10	This study
EG1.2x	EG1 mutant grew on M9 without pyridoxine, isolate E7D11	This study
EG2.1t	EG2 mutant grew on M9 without pyridoxine, isolate E8E12	This study
EG2.2s	EG2 mutant grew on M9 without pyridoxine, isolate E8H12	This study
EG1.3	EG1 mutant grew on Medium X, isolate E7_1	This study
EG1.4	EG1 mutant grew on Medium X, isolate E7_2	This study
EG1.5	EG1 mutant grew on Medium X, isolate E7_4	This study
EG2.3a	EG2 mutant grew on Medium X, isolate E8_1	This study
EG2.4a	EG2 mutant grew on Medium X, isolate E8_2	This study
EG2.5a	EG2 mutant grew on Medium X, isolate E8_4	This study
pydxnA1	SIJ488 Δ*epd* Δ*thrB* Δ*gabD* Δ*sad::km* Δ*gapA::CAP*, isolate 1	This study
pydxnA2	SIJ488 Δ*epd* Δ*thrB* Δ*gabD* Δ*sad::km* Δ*gapA::CAP*, isolate 2	This study
P3	MG1655 Δ*ppsA* Δ*pck* Δ*gcl* Δ*glyA* Δ*pgk::Km*	[21]
P3P	MG1655 Δ*ppsA* Δ*pck* Δ*gcl* Δ*glyA* Δ*pgk* Δ*glcDEF* Δ*patZ*	This study
P3Pe	Evolved P3P harboring plasmids pMP264 and pMP265 grew without glycolate or glycerate	This study
P3Pe Δ*sad*	P3Pe Δ*sad*, cured plasmids	This study

Supplementary Table 2. ***Breseq* identified sequence deviations of the parental (EG1 and EG2) and evolved Δ*epd* Δ*gapA* strains from the SIJ488 reference genome.** Showing mutations with high coverage to support. Gene amplification was manually annotated from the type of "New Junction" predicted by *breseq* and increased coverage. Genomic context of the amplified region are shown in Supplementary Figure 3a. Note that a T172I mutation in *garP* was inadvertently introduced to both parental strains during strain construction.

Position	EG1	EG1.2x	EG2	EG2.1t	EG2.2s	EG2.3a	EG2.4a	EG2.5a	Annotation	Gene	Description
1,750		G→A							G472S (GGC→AGC)	*thrA* →	Bifunctional aspartokinase/homoserine dehydrogenase 1
2,712				Δ1 bp					coding (2376/2463 nt)	*thrA* →	Bifunctional aspartokinase/homoserine dehydrogenase 1
1,608,905		amplification							intergenic (-17/+210)	*uxaB/yneF*	region of [*uxaB/yneF, yneG, glsB, sad, yneJ*]
1,613,342									coding (331/882 nt)	*yneJ*	
1,610,993						amplification	amplification	amplification	coding (467/927 nt)	*glsB*	region of [*glsB, sad, yneJ, yneK, ydeA, marC, marR/mar*]
1,617,763									intergenic (+1/-19)	*marR/marA*	
1,612,127					T→C				Q262R (CAG→CGG)	*sad* ←	succinate semialdehyde dehydrogenase, NAD(P)+-dependent
1,860,978	knock out	knock out	knock out	knock out	knock out	knock out	knock out	knock out	knock out	*gapA*	glyceraldehyde-3-phosphate dehydrogenase A
3,072,093	knock out	knock out	knock out	knock out	knock out	knock out	knock out	knock out	knock out	*epd*	D-erythrose 4-phosphate dehydrogenase
3,273,799	G→A	G→A	G→A	G→A	G→A	G→A	G→A	G→A	T172I (ACT→ATT)	*garP* ←	putative (D)-galactarate transporter

Supplementary Table 3. Statistics of the amplifications of *sad* region shown in Supplementary Figure 3a. Source data are provided as a Source Data file.

Strain	Average coverage			Fold-change[(a)]	Size of amplified region (kb)	Calculated chimeric reads frequency[(b)]	Chimeric reads frequency	Deduced copy number[(c)]
	Up stream	Amplified region	Down stream					
EG2.3a	34	2411	29	76	6.8	98.7%	98.7%	77
EG2.4a	28	2032	20	85	6.8	98.8%	99.0%	100
EG2.5a	53	4090	42	87	6.8	98.8%	98.9%	91
EG1.2x	90	2770	77	33	4.4	97.0%	96.8%	31

[(a)] Coverage ratio of the amplified region to average of up & down stream region. Indicating copy number of the amplified region.

[(b)] Frequency of chimeric reads calculated from "Fold-change" values.

[(c)] Copy number calculated from frequency of chimeric reads mapping to the breakpoints, exported from *breseq*[11,22].

In [(b)] and [(c)], the calculation used formula: $frequency = \frac{copy\ number-1}{copy\ number}$

Supplementary Table 4. X-ray data collection and structure refinement statistics.

SAD variant	WT	WT	Q262R	Q262R
Ligand	NAD^+	NAD^+ & SSA	NAD^+	NAD^+ & SSA
PDB accession	8QMQ	8QMR	8QMS	8QMT
**Data collection**				
Beamline	PETRA III - P14	ID30A-3 (MASSIF-3)	PETRA III - P14	PETRA III - P13
Wavelength (Å)	0.9763	0.9677	0.9763	0.9763
Space Group	$P\,2_1\,2_1\,2_1$	$P\,2_1\,2_1\,2_1$	$P\,2_1\,2_1\,2_1$	$P\,2_1\,2_1\,2_1$
Unit cell dimensions				
a, b, c (Å)	92.1, 116.4, 180.1	91.5, 115.6, 179.6	91.3, 115.7, 179.6	91.7, 115.1, 179.4
α, β, γ (°)	90.0, 90.0, 90.0	90.0, 90.0, 90.0	90.0, 90.0, 90.0	90.0, 90.0, 90.0
Resolution (Å)	19.92 - 1.70 (1.76 - 1.70)	38.07 - 2.30 (2.38 - 2.30)	29.46 - 1.90 (1.97 - 1.90)	29.55 - 1.80 (1.86 - 1.80)
Unique reflections	211047 (20677)	85212 (8417)	149467 (14751)	174478 (17133)
Multiplicity	13.7 (13.5)	8.4 (8.4)	13.8 (13.8)	13.9 (13.9)
Completeness (%)	99.6 (98.6)	99.7 (99.8)	99.3 (99.2)	98.5 (98.4)
I / σI	18.6 (3.1)	9.6 (2.3)	13.4 (2.7)	16.8 (2.3)
R_{merge}	0.090 (0.920)	0.154 (0.973)	0.132 (0.954)	0.098 (1.488)
R_{pim}	0.025 (0.258)	0.055 (0.351)	0.037 (0.264)	0.027 (0.409)
$CC_{1/2}$	0.999 (0.937)	0.997 (0.871)	0.999 (0.930)	0.999 (0.883)
**Refinement**				
R_{work} / R_{free}	0.1692 / 0.1868	0.1962 / 0.2278	0.1813 / 0.2133	0.1802 / 0.2054
RMS bonds	0.006	0.003	0.004	0.006
RMS angles	0.792	0.540	0.728	0.871
Ramachandran				
favored (%)	97.30	97.81	97.54	97.98
allowed (%)	2.70	1.97	2.35	1.86
outliers (%)	0.00	0.22	0.11	0.16
Rotamer outliers (%)	0.58	0.58	0.51	0.51
Number of atoms	15709	14569	15217	15410
Protein	13934	13904	13912	13912
Ligands	176	204	176	204
Solvent	1599	461	1129	1294
Average B-factor	24.49	38.27	28.72	32.48
Protein	23.66	38.29	28.38	31.76
Ligands	24.51	38.67	29.13	32.42
Solvent	31.66	37.53	32.88	40.15

Note that SSA, succinate semialdehyde, has the PDB ligand ID SSN.

Values in parentheses correspond to the highest resolution shell.

Supplementary Table 5. Plasmids used in the study.

Plasmids	Genotype	Source
pET(16b)	pBR322 ori, Amp^R, T7 promoter	Lab collection
pKD3	R6K γ, FRT-CAP-FRT, CAP^r and Amp^r	[23]
pKD4	R6K γ, FRT-Km-FRT, Kan^r and Amp^r	[23]
pTE3842	pET(16b)::*thrA*	This study
pTE3843	pET(16b)::*thrA* G472S	This study
pTE3844	pET(16b)::*thrA* del2376G	This study
pTE3845	pET(16b)::*sad*	This study
pTE3846	pET(16b)::*sad* with Q262R mutation	This study
pCA24N-gabD	pCA24N::*gabD*	ASKA collection[24]
pCA24N-epd	pCA24N::*epd*	ASKA collection[24]
pTE3868	pLac ev: CloDF13 ori, $Spec^R$, $P_{LlacO\text{-}1}$ promoter; empty vector	This study
pTE3827	pLac::*sad*	This study
pTE3828	pLac::*sad* with Q262R mutation	This study
pTE3858	pLac::*thrB*	This study
pTE3867	pLac::*gabD*	This study
pMP264	pSEVA221 RK2, Gm^R pTet-rbsC-ACS_V379A-rbsC-MCRca	This study
pMP265	pSEVA438, pBBR1, $Spec^R$, xylS-Pm, PccBA (M4)	This study

Supplementary Table 6. ***Breseq* identified sequence deviations of the parental (P3P) and evolved (P3Pe) strains from the reference MG1655 genome and plasmids.**
Showing mutations with high coverage to support.

Seq ID	Position	P3P	P3Pe	Annotation	Gene	Description
Genome	257,908	Δ776 bp	Δ776 bp		*[crl]*	[crl]
Genome	481,106	IS5 (+) +4 bp	IS5 (+) +4 bp	intergenic (-398/+145)	*tomB* ← / ← *acrB*	Hha toxicity attenuator; conjugation-related protein/multidrug efflux system protein
Genome	533,853	knock out	knock out	knock out	*gcl*	glyoxylate carboligase
Genome	1,196,220	Δ15,204 bp	Δ15,204 bp		*ymfD, ymfE, lit, intE, xisE, ymfH, ymfI, ymfJ, ymfK, ymfT, ymfL, ymfM, oweE, ymfN, aaaE, ymfR, beeE, jayE, ymfQ, ycfK, tfaP, tfaE, stfE, pinE, mcrA*	cryptic prophage e14
Genome	1,397,381	Δ13,756 bp	Δ13,756 bp		*[ynaJ]–[ttcA]*	Knock-out of [*ynaJ*], *uspE*, *fnr*, *ogt*, *abgT*, *abgB*, *abgA*, *abgR*, *smrA*, *ydaM*, *ydaN*, *dbpA*, [*ttcA*]
Genome	1,615,093		A→C	E97A (GAA→GCA)	*yneJ* →	Transcriptional regulator
Genome	1,756,599	C→A	C→A	A301D (GCC→GAC)	*pykF* →	pyruvate kinase I
Genome	1,784,674	knock out	knock out	knock out	*ppsA*	phosphoenolpyruvate synthetase
Genome	1,978,503		Δ776 bp		*insB1–insA*	*insB1, insA*
Genome	2,173,365	Δ2 bp	Δ2 bp	pseudogene (913-914/914 nt)	*gatC* ←	pseudogene, galactitol-specific enzyme IIC component of PTS
Genome	2,684,204	knock out	knock out	knock out	*glyA*	serine hydroxymethyltransferase
Genome	2,719,896	knock out	knock out	knock out	*patZ*	peptidyl-lysine N-acetyltransferase
Genome	2,861,093	IS4 (+) +13 bp	IS4 (+) +13 bp	coding (130-142/768 nt)	*ygbI* ←	DeoR family putative transcriptional regulator
Genome	3,057,257		G→A	A385V (GCC→GTC)	*serA* ←	D-3-phosphoglycerate dehydrogenase
Genome	3,071,405	knock out	knock out	knock out	*pgk*	phosphoglycerate kinase
Genome	3,124,213	knock out	knock out	knock out	*glcFED*	glycolate dehydrogenase
Genome	3,532,742	knock out	knock out	knock out	*pck*	phosphoenolpyruvate carboxykinase
Genome	3,560,455	+G	+G	intergenic (-2/+1)	*glpR* ← / ← *glpR*	pseudogene becomes intact: Anaerobic respiration; repressor of the glp operon
Genome	4,296,381	+GC	+GC	intergenic (+587/+55)	*gltP* → / ← *yjcO*	glutamate/aspartate:proton symporter/Sel1 family TPR-like repeat protein
pMP265	1,940		Δ4,213 bp		*pccB-pccA*	Knock-out *pccB* and *pccA*

Supplementary Table 7. Specifications of protein purification conditions. Additives to media and buffers are indicated. Otherwise base buffers are listed in the table note.

Protein	Plasmid	Media	IPTG	Lysis buffer	Buffer A	Buffer B	Column	Buffer D
Sad	pTE3845	-	None	-	-	-	Protino	-
Sad Q262R	pTE3846	-	None	-	-	-	Protino	-
GabD	pCA24N-gabD	-	0.25 mM	-	-	-	HisTrap	-
Epd	pCA24N-epd	-	None	-	-	-	Protino	-
ThrA	pTE3842	+ 2 mM $MgSO_4$	None	+ 5 mM βME, 5% glycerol, 1 mM Thr, 5 mM $MgCl_2$	+ 2 mM βME, 5% glycerol, 1 mM Thr, 2 mM $MgCl_2$	+ 2 mM βME, 5% glycerol, 1 mM Thr, 2 mM $MgCl_2$	HisTrap	+ 2 mM βME, 5% glycerol, 1 mM Thr, 5 mM $MgCl_2$
ThrA G472S	pTE3843	+ 2 mM $MgSO_4$	0.2 mM	+ 5 mM βME, 5% glycerol, 1 mM Thr, 5 mM $MgCl_2$	+ 2 mM βME, 5% glycerol, 1 mM Thr, 2 mM $MgCl_2$	+ 2 mM βME, 5% glycerol, 1 mM Thr, 2 mM $MgCl_2$	HisTrap	+ 2 mM βME, 5% glycerol, 1 mM Thr, 5 mM $MgCl_2$
ThrA truncated	pTE3844	-	None	-	-	-	Protino	-

Lysis buffer: 50 mM HEPES-NaOH, 500 mM NaCl, pH 7.5

Buffer A: 50 mM Tris-HCl, 500 mM NaCl, pH 7.5

Buffer B: 50 mM Tris-HCl, 500 mM NaCl, 500 mM imidazole, pH 7.5

Buffer D: 50 mM Tris-HCl, 50 mM KCl, pH 7.5

βME: β-mercaptoethanol; Thr: threonine.

Supplementary Table 8. Specifications of instrument set-up for erythronate, diaminopemelate (DAP), lysine (LYS) and homoserine (HSER) determinations.

Compound name*	Precurser ion (*m/z*)	Product ion (*m/z*)	Dwell time (msec)	Fragmentor voltage (V)	Cell accelerator (V)	Collision energy (eV)	Polarity
Erythronate (Quant)	135.0	75.0	95	380	5	12	Negative
Erythronate (Qual)	135.0	75.0	95	380	5	20	Negative
Erythronate (Qual)	135.0	75.0	95	380	5	20	Negative
DAP (Quant)	191.1	128.0	150	380	5	12	Positive
DAP (Qual)	191.1	81.9	150	380	5	26	Positive
LYS (Quant)	147.1	130.1	150	380	5	8	Positive
LYS (Qual)	147.1	84.1	150	380	5	19	Positive
HSER (Quant)	120.1	74.0	150	380	5	10	Positive
HSER (Qual)	120.1	56.1	150	380	5	20	Positive

*: Quant and Qual in parentheses correspond to quantification fragment and qualification fragment, respectively.

Supplementary references

1. Jensen, S. I., Lennen, R. M., Herrgard, M. J. & Nielsen, A. T. Seven gene deletions in seven days: Fast generation of *Escherichia coli* strains tolerant to acetate and osmotic stress. *Sci Rep* **5**, 17874 (2015).
2. Hayashi, K. *et al*. Highly accurate genome sequences of *Escherichia coli* K-12 strains MG1655 and W3110. *Mol. Syst. Biol.* **2**, 2006.0007 (2006).
3. Murigneux, V. *et al.* MicroPIPE: validating an end-to-end workflow for high-quality complete bacterial genome construction. *BMC Genomics* **22**, 474 (2021).
4. Kolmogorov, M., Yuan, J., Lin, Y. & Pevzner, P. A. Assembly of long, error-prone reads using repeat graphs. *Nat. Biotechnol.* **37**, 540–546 (2019).
5. Otto, T. D., Dillon, G. P., Degrave, W. S. & Berriman, M. RATT: Rapid Annotation Transfer Tool. *Nucleic Acids Res.* **39**, e57 (2011).
6. Scheffen, M. *et al.* A new-to-nature carboxylation module to improve natural and synthetic CO_2 fixation. *Nat. Catal.* **4**, 105–115 (2021).
7. Grant, G. A., Hu, Z. & Xu, X. L. Identification of amino acid residues contributing to the mechanism of cooperativity in *Escherichia coli* D-3-phosphoglycerate dehydrogenase. *Biochemistry* **44**, 16844–16852 (2005).
8. Bell, J. K., Pease, P. J., Bell, J. E., Grant, G. A. & Banaszak, L. J. De-regulation of D-3-phosphoglycerate dehydrogenase by domain removal. *Eur. J. Biochem.* **269**, 4176–4184 (2002).
9. Oberhardt, M. A. *et al.* Systems-wide prediction of enzyme promiscuity reveals a new underground alternative route for pyridoxal 5'-phosphate production in *E. coli*. *PLoS Comput. Biol.* **12**, e1004705 (2016).
10. Richts, B. & Commichau, F. M. Underground metabolism facilitates the evolution of novel pathways for vitamin B6 biosynthesis. *Appl. Microbiol. Biotechnol.* **105**, 2297–2305 (2021).
11. Barrick, J. E. *et al.* Identifying structural variation in haploid microbial genomes from short-read resequencing data using *breseq*. *BMC Genomics* **15**, 1039 (2014).
12. Jumper, J. *et al.* Highly accurate protein structure prediction with AlphaFold. *Nature* **596**, 583–589 (2021).
13. Varadi, M. *et al.* AlphaFold Protein Structure Database: massively expanding the structural coverage of protein-sequence space with high-accuracy models. *Nucleic Acids Res.* **50**, D439–D444 (2022).
14. Baugh, A. C., Momany, C. & Neidle, E. L. Versatility and complexity: common and uncommon facets of LysR-type transcriptional regulators. *Annu. Rev. Microbiol.* **77**, 317–339 (2023).

15. Mayo-Pérez, S., Gama-Martínez, Y., Dávila, S., Rivera, N. & Hernández-Lucas, I. LysR-type transcriptional regulators: state of the art. *Crit. Rev. Microbiol.* **0**, 1–33 (2023).
16. Schuller, D. J., Grant, G. A. & Banaszak, L. J. The allosteric ligand site in the V_{max}-type cooperative enzyme phosphoglycerate dehydrogenase. *Nat. Struct. Biol.* **2**, 69–76 (1995).
17. Monterrubio, R., Baldoma, L., Obradors, N., Aguilar, J. & Badia, J. A Common regulator for the operons encoding the enzymes Involved in D-galactarate, D-glucarate, and D-glycerate utilization in *Escherichia coli*. *J. Bacteriol.* **182**, 2672–2674 (2000).
18. Beber, M. E. *et al.* eQuilibrator 3.0: a database solution for thermodynamic constant estimation. *Nucleic Acids Res.* **50**, D603–D609 (2022).
19. The UniProt Consortium *et al.* UniProt: the Universal Protein Knowledgebase in 2023. *Nucleic Acids Res.* **51**, D523–D531 (2023).
20. Price, M. N. & Arkin, A. P. A fast comparative genome browser for diverse bacteria and archaea. Preprint at https://doi.org/10.1101/2023.08.23.554478 (2023).
21. Aslan, S., Noor, E., Vaquerizo, S. B., Lindner, S. N. & Bar-Even, A. Design and engineering of *E. coli* metabolic sensor strains with a wide sensitivity range for glycerate. *Metab. Eng.* **57**, 96–109 (2020).
22. Deatherage, D. E. & Barrick, J. E. Identification of Mutations in Laboratory-Evolved Microbes from Next-Generation Sequencing Data Using *breseq*. in *Engineering and Analyzing Multicellular Systems* (eds. Sun, L. & Shou, W.) vol. 1151 165–188 (Springer New York, New York, NY, 2014).
23. Datsenko, K. A. & Wanner, B. L. One-step inactivation of chromosomal genes in *Escherichia coli* K-12 using PCR products. *Proc. Natl. Acad. Sci. U. S. A.* **97**, 6640–5 (2000).
24. Kitagawa, M. *et al.* Complete set of ORF clones of *Escherichia coli* ASKA library (A Complete Set of *E. coli* K-12 ORF Archive): Unique Resources for Biological Research. *DNA Res.* **12**, 291–299 (2006).
