# Supplementary material for: Adaptive laboratory evolution recruits the promiscuity of succinate semialdehyde dehydrogenase to repair different metabolic deficiencies
Source: Nat Commun. 2024 Oct 15;15:8898. doi: 10.1038/s41467-024-53156-x (PMC11480449; doi:10.1038/s41467-024-53156-x)
Supplement: Supplementary file 3 — Description of Additional Supplementary Files [file 41467_2024_53156_MOESM3_ESM.pdf]

Description of Additional Supplementary Files

File Name: Supplementary Data 1

Description: Oligo primers used in the study.
